# Enhancing the properties of geopolymer concrete using nano-silica and microstructure assessment: a sustainable approach

**DOI:** 10.1038/s41598-023-44491-y

**Published:** 2023-10-12

**Authors:** Koti Chiranjeevi, Marykutty Abraham, Badrinarayan Rath, T. R. Praveenkumar

**Affiliations:** 1 Department of Civil Engineering, Sathyabama Institute of Science and Technology, Chennai, India; 2 Center for Remote sensing & Geoinformatics, Sathyabama Institute of Science and Technology, Chennai, India; 3 Department of Civil Engineering, National Institute of Technology Mizoram, Aizawl, India; 4 Department of Construction Technology and Management, Wollega University, Nekemte, Ethiopia

**Keywords:** Civil engineering, Energy infrastructure

## Abstract

Nowadays low calcium fly ash-based geopolymer concrete can be replaced with cement-based concrete to avoid the adverse effect of manufacturing cement on the environment. Utilization of geopolymer concrete instead of traditional concrete using low calcium fly ash and nano silica reduces a significant amount of CO­_2_ emission towards the atmosphere. However, the performance of geopolymer concrete is less than that of Portland cement concrete. To improve the performance of geopolymer concrete nano silica was used in the present study. In this work, geopolymer concrete was made utilizing fly ash, ground granular blast furnace slag (GGBS), and sugarcane bagasse ash. In the first instance, binary combinations i.e. fly ash and GGBS were employed as cementitious materials for the production of geopolymer concrete. In the second instance, a ternary mixture of pozzolanic material was prepared by taking 25% GGBS, 65% Fly ash, and 10% bagasse ash. In the third instance, varying percentages of nanoparticles were used for the above ternary mixture. The mechanical and durability properties of the geopolymer composite that was made earlier were tested. The compressive strength and split tensile strength of geopolymer composites were assessed for mechanical properties and a rapid chloride permeability test, water absorption test, and acid attack test were done to know about the porosity of concrete. Results showed that, with a dose of 4% nanoparticles, the durability and strength properties of the concrete had improved the most. The GCBA-N4 mixture had the highest split tensile and compressive strength was measured to be 2.91 MPa and 41.33 MPa and the rapid chloride permeability test, water absorption rate, and percentage of mass loss due to sulfate attack were found as a minimum for GCBA-N4 specimen.

## Introduction

Geopolymer concrete is considered the next generation of concrete since it eliminates the need for standard Portland cement in its production, hence reducing CO_2_ emissions. A solution of sodium hydroxide, sodium silicate, and sodium hydroxide or water is used to activate sodium aluminosilicates such as fly ash, granulated blast furnace slag, metakaolin, and volcanic ash to form geo-polymer concrete (GPC). Using geopolymer concrete composites can reduce the amount of heat needed to form early concrete strength, as well as the amount of fly ash and slag cement that must be disposed of in landfills, as well as the amount of CO_2_ emissions generated by such landfills. Cement paste and aggregates are the primary ingredients in concrete, a cementitious composite. Conventional concrete manufacturing relies heavily on Portland Cement (PC)^1^. A ton of Portland Cement produces 600 kg to 800 kg of Carbon dioxide based on the amount of CO_2_ generated per ton of cement produced^2^. It has also been demonstrated that the price of geopolymer concrete is about the same as that of Ordinary Portland Cement concrete while lowering emissions of carbon dioxide by 22–72%. While using geopolymer concrete instead of Ordinary Portland Cement composites might increase sustainability, geopolymer concrete has proved to have good strength and durability qualities.

As a result of the high expenses and health risks connected with the disposal of agricultural and industrial wastes such as fly ash, rice husk ash and GGBS in recent years, recycling and reusing of these wastes has become an immensely complicated issue to be addressed. Although their use in cement manufacturing has helped to improve the system performance over the past several decades, the growing demand for the production of cement on a huge scale has piqued the interest of researchers who are concerned about the influence of carbon dioxide emissions on global warming. This represents around 5–8% of overall CO_2_ emissions into the atmosphere, according to the Environmental Protection Agency (EPA)^3–5^. In recent years, geopolymer materials have been the focus of the majority of the study. Geopolymer is an environmentally friendly and highly efficient construction binding material that offers significant environmental benefits over other binding compounds. It is made by reacting with an alumino-silicate source in a high pH environment, which has significant environmental benefits over typical binding chemicals^6–9^. For the first time, a new adhesive compound that may be made at low or moderate temperatures has been referred to as “Geopolymer” by Davidovits. It is now the primary focus of geopolymer research and development to create alternative cement manufacturing techniques and cost-effective construction materials. Because geopolymer materials have a lower calcination temperature than conventional procedures, they emit significantly less CO_2_ than conventional processes.

GGBS is an industrial by-product, produced during the production of steel and iron in the blast furnace, where iron ore (Fe_3_O_4_, Fe_2_O_3_, and so on), coke, and CaCO_3_, are heated to a particular temperature of approximately 1500 °C. Since these materials soften in the blast furnace, molten irons are separated from the impurities in the melting process. Those impurities are known as slag. Molten slags are lighter than molten iron, and so molten slags float above the molten iron and are quickly cooled with a high-pressure water jet to form granular particles before being cooled further. After cooling they are fed to a ball mill and ground into a fine powder to produce GGBS^10–15^.

In the construction industry, GGBS is a well-known building material that is used to prepare high-quality blended mortar, cement, and concrete. Using GGBS can be reduced the amount of OPC in concrete production by around 35–70%^16–20^. When they are finely powdered, it has been demonstrated as outstanding capabilities to play the role of cementitious materials. Due to this, it can be blended with some other alkali activated materials to produce geopolymer concrete. The ground granules blast furnace slag contains mono-silicates, which are similar to the mono-silicates present in Ordinary Portland Cement clinker and dissolve in any media once the mixing process has started. By using GGBS in geopolymer concrete the formation of voids can be reduced. Furthermore, it has the potential to improve the resistance of concrete against sulfate and alkali-silica reactions by lowering the requirement for water during the curing process^21–25^. Taking all of these advantages into consideration, it appears to be a strong candidate for inclusion in the GPC.

Now a days, most of the researchers are using nanomaterials in concrete for making it high performance concrete economically and ecologically way. The nanomaterials are several types such as nano-TiO_2_, nano Al_2_O_3_, nano SiO_2_, nano-Fe_2_O_3_, carbon nanotubes etc. Among them, nano silica is being used widely in cement and concrete for improving their performance. Because it plays a dual role i.e. participating in the pozzolanic activity and improving the packing density^26^. When nano silica was incorporated into concrete, had shown higher compressive strength^27^, increased tensile strength^28^ and bending strength^29^. Also, many researchers had reported about the permeability properties of nano silica concrete that the concrete had low capillary absorption, water absorption and both liquid and gas permeability as compared to normal concrete^30–32^. Also, the combined addition of nano silica and GGBS to the concrete improved the rate of hydration and tensile strength^33^. When nano silica was added to high volume fly ash and slag concrete, there was a reduction in the initial and final setting time of the concrete, chloride ion penetration, and length of the dormant period during the hydration process^34,35^. Also, the addition of nano silica to fly ash cement mortar improved the early age strength but reduced the later age strength^36^. The cement was replaced with high volume glass waste glass powder with nano silica and prepared the concrete^37^. The concrete in which cement was replaced by waste material had low strength and delay in setting time, but this problem was resumed by the addition of nano silica in that concrete^38^. Also, it was found that the addition of nano silica in oil well cement improved the strength and reduced the setting time^39^.

Geopolymer concrete is an eco-friendly and innovative building material in which the cement is completely replaced by waste pozzolanic materials. Many promising results were shown by conducting a lot of research on high-performance geopolymer concrete, which will give benefit the field of construction engineering. But the utilization of high-performance geopolymer concrete is still in paperwork in most African Countries due to several factors. They may be (i) difficulties for the production of fined pozzolanic materials, (ii) non-availability of mix design process, (iii) non-establishment of practical methods in the field, (iv) not properly utilization of base material, (v) non-availability of equipment for the production of high-performance geopolymer concrete. The above difficulties can be solved by utilizing nano materials in the production of geopolymer to make it efficient. Limited research has been carried out in which nanoparticle are used in geopolymers to increase their performance. Also, many researchers have prepared geopolymer concrete by using a unary or binary combination of pozzolanic materials. But, in the present research, the geopolymer concrete has been prepared by a ternary combination of pozzolanic materials named sugar cane bagasse ash, fly ash and GGBS. Also, limited research had been carried out on the strength and durability of geopolymer concrete with the addition of nano silica. In this research, nano silicas were selected to mix with pozzolanic materials of geopolymer concrete to increase the performance. Various strength and durability properties were checked for new mixes of geopolymer concrete. It was expected that the specific ultra-dense volume, as well as packing density of geopolymer concrete, might be increased due to the addition of nano silica. It will helpful to increase the performance of geopolymer concrete, particularly its strength and durability. It can be noticed that they are very fine particles with granular shapes. Hence, the stiffness of geopolymer concrete might be increased resulting reduction in cracks in concrete structure due to the fineness property of the nano silica. The novelty of the present research is the influence of nano silica on the strength and durability of ternary blending pozzolanic material geopolymer concrete. The objectives of the present research are:

(i) To investigate the performance of nano silica on the workability of ternary blended geopolymer concrete.
(ii) To investigate the performance of nano silica on the strength of ternary blended geopolymer concrete.
(iii) To investigate the performance of nano silica on the durability of ternary blended geopolymer concrete.

## Methodology

**Figure 1:**
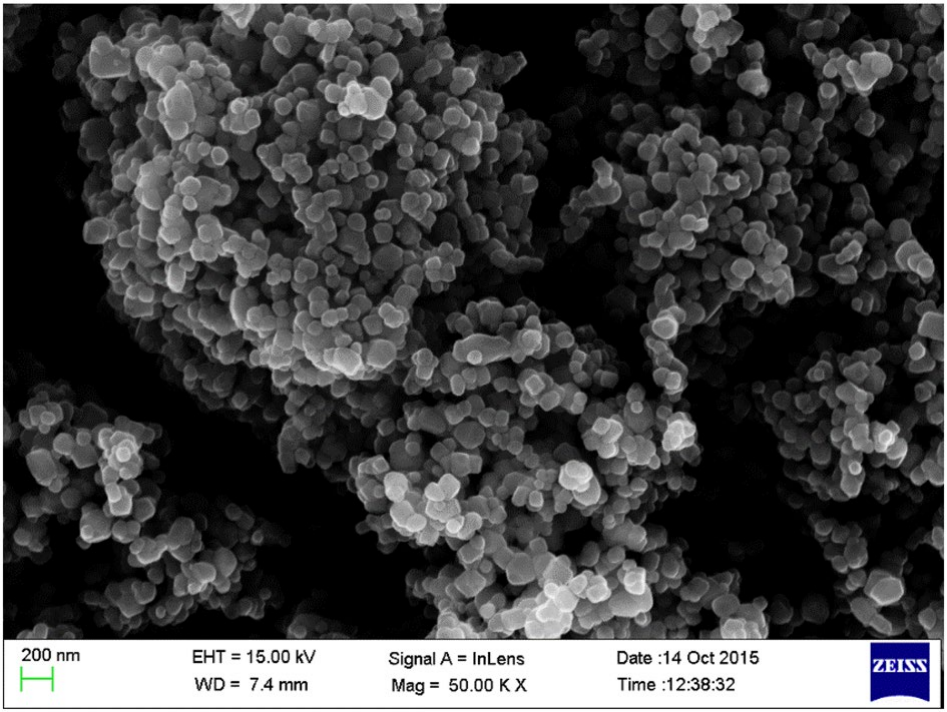
SEM image of nano silica.

Within the scope of this study, eight different concrete mixes were prepared to explore the influence of the nanomaterial and sugarcane bagasse ash (SCBA) on the mechanical properties of the blended geopolymer concrete (mixing of sugar cane bagasse ash, fly ash and GGBS). Natural fine and coarse aggregates, cementitious materials produced from an alumino-silicate source, a pozzolanic mixture of 65% fly ash (FA), 25% ground granular blast furnace slag (GGBS) and 10% bagasse ash, an alkaline activator, were the main ingredients for the preparation of geopolymer concrete. Mixes that had been combined with nano silica of varying proportions ranging from 0 to 6% as an additive to cement. The granular size of nano silica was 10^–9^ m and its SEM image has been shown in Fig. 1. NaOH and CaSiO_3_ were used as an activator to prepare the geopolymer. The NaOH is available in the market in tablet form. The sodium hydroxide solution was prepared 24 h before the production of concrete by mixing 42 g of NaOH with 1 L water. The ratio between NaOH and CaSiO_3_ was 1:2.5 the ratio between alkali activator solution to ternary blended pozzolanic material was 1:0.35. A homogenizing procedure was used to improve the dispersion of nanoparticles in the solution. Table 1 shows the mix proportions of geopolymer concrete mixes that have been specifically prepared. After the dry mixing of binary and ternary blended pozzolanic materials with fine aggregate and coarse aggregate, the prepared sodium hydroxide solution and calcium silicate gel were poured to the dry mix. The slump cone test was conducted after mixing all ingredients in the fresh stage. The required cubes and cylinders of the desired size were cast after conducting the slump cone test. After 24 h all the cubes and cylinder samples were remolded and kept in the oven for upto 90 days at 105 degrees centigrade. Three samples were cast for each mix of concrete for conducting laboratory tests that correspond to thermal curing days. Several tests were conducted on both fresh concrete and hardened concrete to observe the performance of nano particles in concrete. The objective of the laboratory test and the total number of samples for the corresponding laboratory test were described in Table 2.

**Table 1:** Mix proportions of ingredients for production of 1 m^3^ geopolymer concrete.

Sample ID	Flyash (kg)	GGBS (kg)	SCBA (kg)	FA (kg)	CA (kg)	Nano silica (kg)	NaOH (kg)	CaSiO_3_ (kg)	Water (Lit)
GCC	303.5	101.25	0	595	1176	0	42	105	1009
GCBA-N0	263	101.25	40.5	595	1176	0	42	105	1009
GCBA-N1	263	101.25	40.5	595	1176	4.05	42	105	1009
GCBA-N2	263	101.25	40.5	595	1176	8.1	42	105	1009
GCBA-N3	263	101.25	40.5	595	1176	12.15	42	105	1009
GCBA-N4	263	101.25	40.5	595	1176	16.2	42	105	1009
GCBA-N5	263	101.25	40.5	595	1176	20.25	42	105	1009
GCBA-N6	263	101.25	40.5	595	1176	24.3	42	105	1009

**Table 2:** Objectives of laboratory test and numbers of corresponding samples.

Sr. no	Experiment	Objective	Type of sample	No. of samples
1	Slump cone test	To measure the concrete consistency between the mixed prepared in laboratory. To investigate the behavior of compacted concrete cone under the action of gravitational force.	Fresh concrete mix with nano silica at different percentages.	24
2	Compressive strength	To measure the axial compressive load or stress can resist before fracturing for new mixed concrete samples	150 mm × 150 mm × 150 mm cubes were cast and cured up to 90 days	72
3	Split tensile strength	To determine the tensile strength of various mixes in lateral direction under axial compressive load	Cylinders of 150 mm diameter and 300 mm height were cast and cured up to 90 days	72
4	RCPT	To determine the resistance capacity of new mixed concrete against chloride ion penetration	Cylinder of 100 mm diameter and 50 mm height were cast and cured up to 90 days	48
5	Sulphate resistance	To investigate the durability aspect of new mixed concrete containing nano silica in acidic environment	100 mm × 100 mm × 100 mm cubes were cast and cured in water for 28 days followed by immersing in Na_2_SO_4_ upto 90 days	48
6	Water absorption	To measure the water accessible porosity of concrete of different mixes To compare the improvement of microstructure of new mixed concrete indirectly	100 mm × 100 mm × 100 mm cubes were cast and cured in water up to 90 days. The harden concrete cubes were again kept in water for 24 h	48
7	In-elastic neutron scattering (INS)	To get a knowledge about the production of Calcium aluminate silicate hydrate gel (CASH) gel for different mixes of concrete	A small chunk of concrete kept under the spectrometer	8
8	XRD	To determine the various components for better hydration process of concrete	A small amount of powdered concrete has been taken and X-ray was incident on it	8

## Results and discussion

In the present research, the effect of nano-silica and bagasse ash in fly ash-blast furnace slag-based geopolymer concrete on strength and durability had been studied at a deep level. Here two types of materials were introduced. The first one is bagasse ash, which replaced partially the pozzolanic material up to 10% and the second one was the addition of nano-silica externally to those pozzolanic mixtures up to 6%. Where the pozzolanic material used in geopolymer concrete was a binary blending of fly ash and blast furnace slag. Two types of observations were done in this research. The first one was the action of bagasse ash on the pozzolanic activity for the ternary combination of fly ash, GGBS, and bagasse ash and the second one was the playing role of nano-silica on packing density of geopolymer concrete. For gaining the above knowledge both fresh concrete test and harden concrete test were conducted on new mixed geopolymer concrete and the observed results are discussed below.

### Slump and density of designed mixed geopolymer concrete

Relative slump values are based on the flow test with 25 times tamping. This is important to note. Table 3 shows how easily lightweight EGC mixes flow (work) in elements of a relative slump. Regardless of composite type, all of the mixes displayed superior flowability. When Nano-silica and bagasse ash was added to geopolymer mixtures, the relative slumps increased considerably. When the 10% bagasse ash was replaced with the binary blended pozzolanic materials the slump height increased by 52%. This happened due to decreasing the number of flocculated pozzolanic particles of binary blended concrete and increasing the ball-bearing effect inside the geopolymer concrete. But when 1% of nano silica was introduced to the ternary blending of geopolymer concrete, the slump height increased by 72%. This happened due to the increasing packing density. The fined nano silica inserted the gaps of quaternary blended concrete and reduced the porosity value. Due to increasing packing density, the surface of an area of quaternary blended pozzolanic material had decreased and the geopolymer concrete became more workable as compared to ternary mixed concrete. This type of increasing trend continued till the addition of nano-silica up to 4%. At that position, the slump height was measured 2.75 times than the slump height of binary blended concrete. That means increasing the percentage of nano silica increased the packing density and decreased the total surface of the pozzolanic mix, which leads to the flowability of concrete. Also, the spherical granular shape of nano silica provided a ball-bearing action in between the ternary mixed particles, which increased the mobility action during mixing time. Simultaneously it can be noticed that the introduction of bagasse ash and nano-silica decreased the density of concrete gradually as their increasing of percentage. This happened due to the lower specific gravity of both materials as compared to fly ash and GGBS. Lightweight geopolymer composites that had more nanoparticles didn’t flow as compared to the earlier mix, so the relative slump of the mix with 5% nano-silica was 21% less than that of 4% nano-silica concrete, as shown in Table 3. Further addition of nano-silica resulted in the decrease of the slump height. Beyond the 4% of addition of nano-silica might be created extra voids inside the geopolymer concrete. Due to the extra fineness property nano silica are usually able to store more water than other nanoparticles, which could make the mortarless able to flow. This was caused by the nanoparticle’s hydrophilia, which caused water to be absorbed from the composite due to the high surface area of the nanoparticles. The addition of nano silica (NS) also made the EGC-matrix less likely to slump by 8.8%. This is because the surface of NS is very reactive and has a lot more Blaine fineness than other materials, as shown in Table 1. Also, because NS was in the matrix, the nano-silica sol and the pozzolanic materials were in direct contact with each other. Also, the way the nano-silica particles are mixed could be a factor in the loss of flowability because they aren’t all the same size.

**Table 3:** Slump and density of geopolymer specimens.

Mix code	Relative slump	Density (kg/m^3^)
GCC	–	1778
GCBA-N0	1.52	1764
GCBA-N1	1.72	1758
GCBA-N2	2.32	1789
GCBA-N3	2.51	1767
GCBA-N4	2.75	1754
GCBA-N5	2.27	1723
GCBA-N6	2.21	1643

### Compressive strength

**Figure 2:**
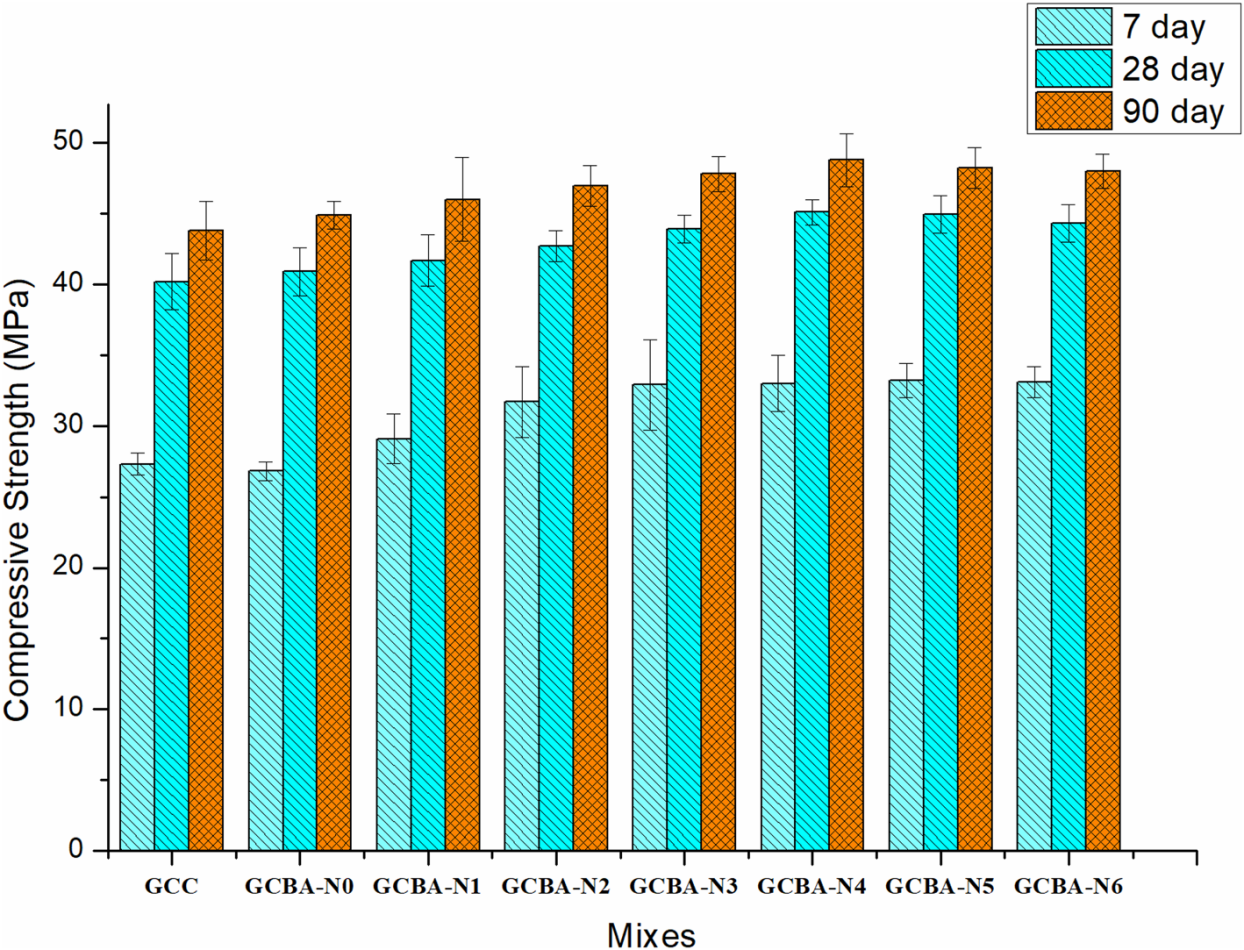
Compressive strength of geopolymer specimens.

Figure 2 depicts the compressive strength of geopolymer concrete specimens with varying percentages of nano silica ranging from 0 to 6% which were cured at 7, 28, and 90 days, respectively. The compressive strength of both ternary blended-based geopolymer concrete with nano silica and without nano silica had increased with the length of time, the concrete had been in place.

Observing the above figures, it can be seen that the compressive strengths of the GPC with BA and GPC conventional are 43.17 MPa and 44.27 MPa, respectively, after 28 days of thermal curing in the laboratory. The compressive strength had risen by 12% after the addition of one percent of nano-silica particles to the mix. The compressive strength had increased by 16.12% with the addition of 3% nano-silica. In a composite, the proportion of nano silica ready for reaction increased as the volume fraction of nanoparticles in the composite increased. The reaction might proceed much more quickly than in mixes that do not contain nano-alumina. According to the researchers, this could be due to the presence of responsive silica, which controlled the polymerization reaction and promoted the formation of an alumina silica gel, which was required for the nanocomposite’s material strength development^15,40^. The formation of C-A-S-H gel took place to a greater extent in the ternary and quaternary blended geopolymer concrete as compared to binary blended geopolymer concrete. The addition of nano-silica helped to form extra C-A-S-H gel by reacting with calcium content in fly ash, resulting in better packing in the matrix. Due to the formation of extra C-A-S-H gel, the microstructure of geopolymer concrete improved. Hence the absorption capacity of the cube had increased by 15%, 12%, and 6% for 7-, 28- and 90-day thermal curing respectively against the compressive strength. From the above results, it was found that the nano-silica had played a better role in gaining the early age strength of concrete.

Increasing the nano-alumina content above the optimum level, on the other hand, results in a reduction in strength. Excess nano-alumina can aggregate and generate faults in the specimen, such as pores or unreacted particles, which are caused by unreacted components remaining in the specimen after the reaction. The responsible factor for starting decrement in compressive strength beyond the addition of nano-silica as 6% might be the replacement of stronger material (fly ash and GGBS) with the weaker material (bagasse ash) and the absence of pozzolanic activity in ternary mixed concrete. Also, it might be happened due to the un-hydrated cement was coated with extra calcium hydroxide, which was produced due to the addition of nano-silica beyond 4%. Hence, the hydration process might be slow and reduce the strength properties of geopolymer concrete.

### Split tensile strength

**Figure 3:**
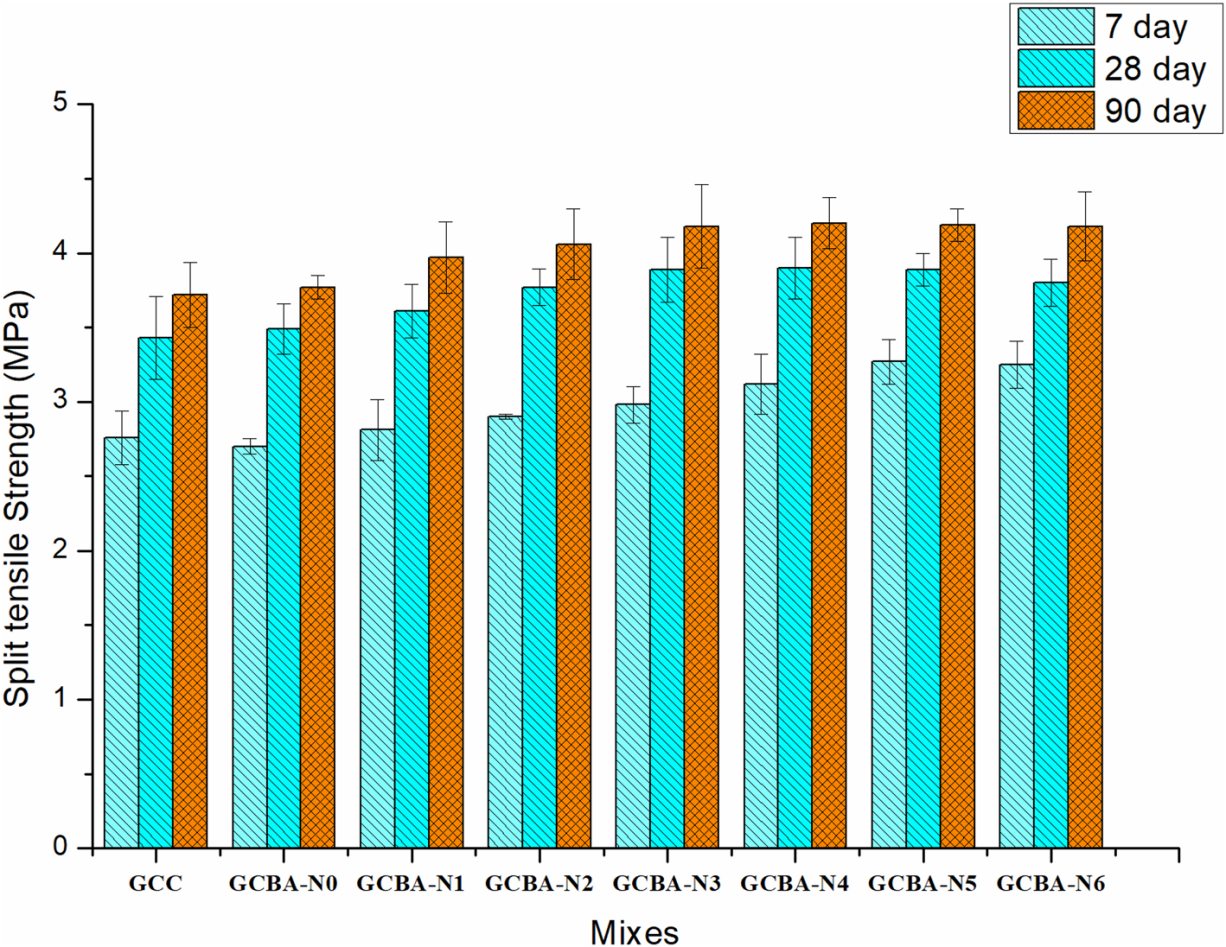
Split tensile strength of geopolymer specimens.

As shown in Fig. 3, the split tensile strengths of GCBA-N0, GCBA-N1, GCBA-N2, GCBA-N3, GCBA-N4, GCBA-N5, and GCBA-N6 samples are 3.51 MPa, 3.64 MPa, 3.78 MPa, 3.92 MPa, 3.89 MPa, 3.85 MPa, and 3.78 MPa, respectively, after 28 days of testing, and they are 2.7 MPa, 2.75 MPa, 2.85 MPa, and 2.9 MPa, 3.1 MPa, 3.0 MPa, and 2.89 MPa after 7 days of testing. The splitting tensile strength of the geopolymer concrete increases in a manner, similar to that of the geopolymer specimens under compression as the dose of nanoparticles is increased. After seven days of testing, the highest improvement in tensile strength of approximately 19.3% is recorded for the GCBA-N4 combination.

According to Fig. 3, when fly ash, bagasse ash, and GGBS are employed in amounts of 65%, 10%, and 25% respectively with varied dosages of nanoparticles, the splitting tensile strength follows the same pattern as that observed previously. The GCBA-N4 sample exhibits the greatest rise in value (65% fly ash, 10% bagasse Ash, 25% GGBS, and 4% nanoparticles). After 7 and 28 days of testing, the strength was increased by 26.78% and 27.6%, respectively. The highest split tensile strength may be attained by preparing the mix with a 3% dosage of the superplasticizer, which is the case in this instance. Zhou et al.^41^ also confirmed similar results that, the tensile strength increased due to the addition of a small amount of nano-silica, but the tensile strength was showing worsen value when higher nano-silica was content. The increase of split tensile strength due to the introduction of nano silica up to 4% and decreased beyond it, happened because of agglomeration. This might be in homogeneity in uncured resin zones, while extra energy was imposed into the mixing process and increased the porosity. By using the nano-silica in geopolymer concrete the water binder ratio was reduced and improved the mortar matrix and interfacial transition zone. This zone became the weakest zone through which cracks was passing through the coarse aggregate particles for the geopolymer concrete in which nano-silica was added more than 4%^34,35^.

### Rapid chloride permeability test

**Figure 4:**
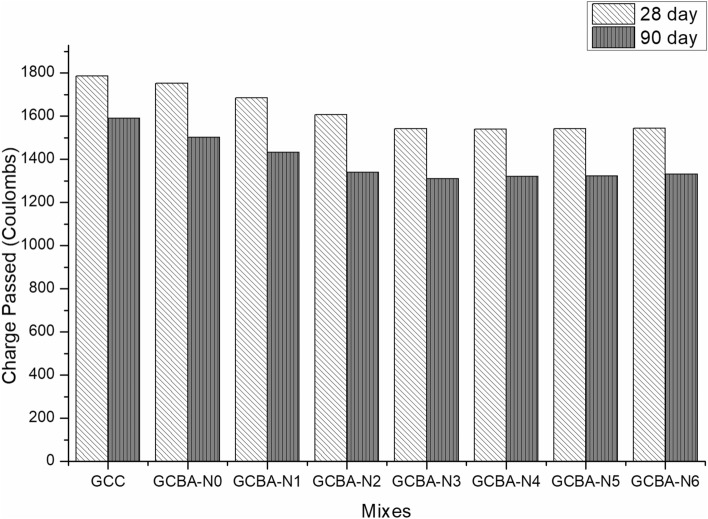
RCPT values of geopolymer specimens.

One of the most important elements influencing concrete durability in corrosive conditions is its resistance to chloride penetration^42^. The standard defines degrees of chloride penetration that are evaluated based on the charge transmitted through the specimens being tested. The results revealed that, in general, replacing the binder with Nanosilica enhanced the resistance of the concrete mix to chloride penetration when compared to the conventional mixture. The charge (coulombs) that passed for the control mix (which did not contain any Nanosilica and bagasse ash) was 1071, indicating a very low chloride penetration level. However, as shown in Fig. 4, the discharged charge (coulombs) for the Nanosilica blended specimens was 972 (which was classified as extremely low). The chloride diffusion resistance rose by 29.17 percentage points as compared to the control mix. This discovery is consistent with the literature, which states that the inclusion of Nanoparticles enhances the chloride diffusion resistance of concrete^16^. The influence of nucleation on nanomaterials is responsible for substantial mechanical improvement. Furthermore, a well-dispersed and homogenizednano dispersion may greatly increase particle packing in geopolymer concrete, resulting in a microstructure that appears extremely compacted and dense. Also, the spherical size of nano silica and bagasse ash might be improved the packing density of geopolymer concrete. The passing of coulomb charges through the surface of geopolymer concrete of 90 days cured was less than the 28 days cured geopolymer concrete for every mix containing nano-silica due to the dense microstructure of concrete. The addition of nano-silica reduced the chloride ion penetration in self-compaction as reported by the previous researcher^43^. This happened due to refined pore structure and densified microstructure. Also, it was reported that the RCPT values of fly ash-based geopolymer mortar with nano silica were reduced due to the presence of more crystalline compounds^44^. These mechanisms halt fracture formation and act as a bridge between them, inhibiting crack growth. This, in turn, increases the material’s resistance to chloride diffusion.

### Water absorption test

**Figure 5:**
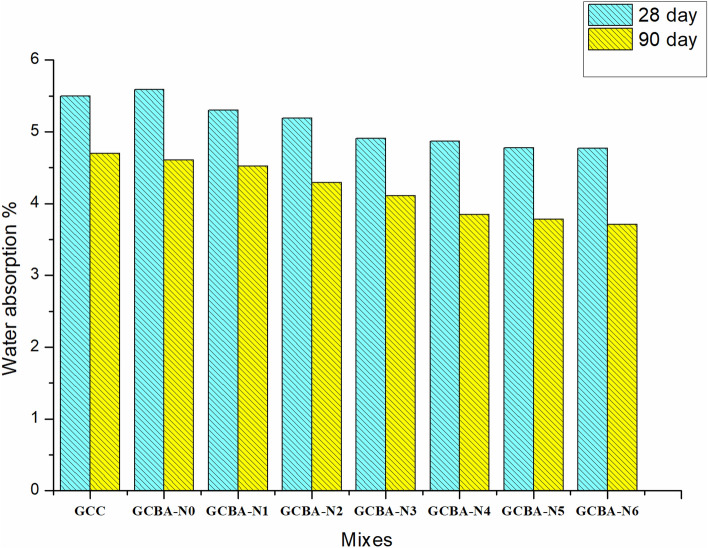
Water absorption % of geopolymer specimens.

When it comes to concrete’s long-term durability, it’s crucial to consider how much water it can absorb. The concrete and structure’s reinforcement both suffer when water gets inside. At present days, water absorption has been considered an important factor for concrete durability, since it measures the transportation of moisture into unsaturated specimens. The water absorption test measures the driving force for the water entry through the concrete surface by both the pressure head and the capillary suction. The water absorption coefficient plays an important role to predict the service life of the concrete structure. The results of a water absorption test are shown in Fig. 5.

Figure 5 clearly shows that water absorption decreased as the quantity of nano-silica with bagasse ash increased. For the binary blending of fly ash and GGBS as a binder, the water absorption was higher than for ternary and quaternary blending with bagasse ash and Nano silica respectively. In the first scenario, the GCBA-N0 mix had a water absorption of 5.5% at 28 days of thermal curing and 4.5% at 90 days of thermal curing cubes. The water absorption percentage for a concrete cube cured by 28 days for sample GCBN-0 was slightly higher than the sample GCC, but for a concrete cube cured by 90 days, the water absorption percentage was reversed. It indicates that the hydration reaction became slow for 28 days of thermal curing when bagasse ash was replaced with the 10% blending of fly ash and GGBS. But after 90 days of thermal curing, the pores inside ternary blending geopolymer concrete were filled with C-A-S-H gel. Hence, the water absorption percentage of 90 days of cured concrete cubes had reduced as compared to 28 days of cured concrete cubes. But when the nano-silica had introduced up to 6% in ternary blending geopolymer concrete, the water absorption capacity gradually reduced for both 28 days and 90 days of cured concrete of every mix. When 6% of nano silica was mixed with ternary blended geopolymer concrete the percentage of water absorption for 28 days and 90 days of thermal curing decreased by 0.5% and 0.8% respectively than controlled geopolymer concrete. The combined action of nano silica and bagasse ash absorbed water in a lower amount due to the attribution of a higher pozzolanic effect, which made the geopolymer concrete denser and more compact. The pore structure of geopolymer concrete was filled by fined-grained nano silica and bagasse ash, which in turn reduced the water absorption of the concrete. Since the nano-silica were fined particles, the nano-silica filled the pores in the bulk pozzolanic paste and reduced the capillary pores. Thus, the addition of nano-silica may be beneficial to reduce the capillary absorption of concrete and gain in strength. Increased nano silica dosage improved the density of geopolymer concrete, which is the primary reason for lower water absorption with higher nano dosages. This means that with only a 6% superplasticizer dosage, the smallest amount of water absorption occurs.

### Sulphate resistance test

**Figure 6:**
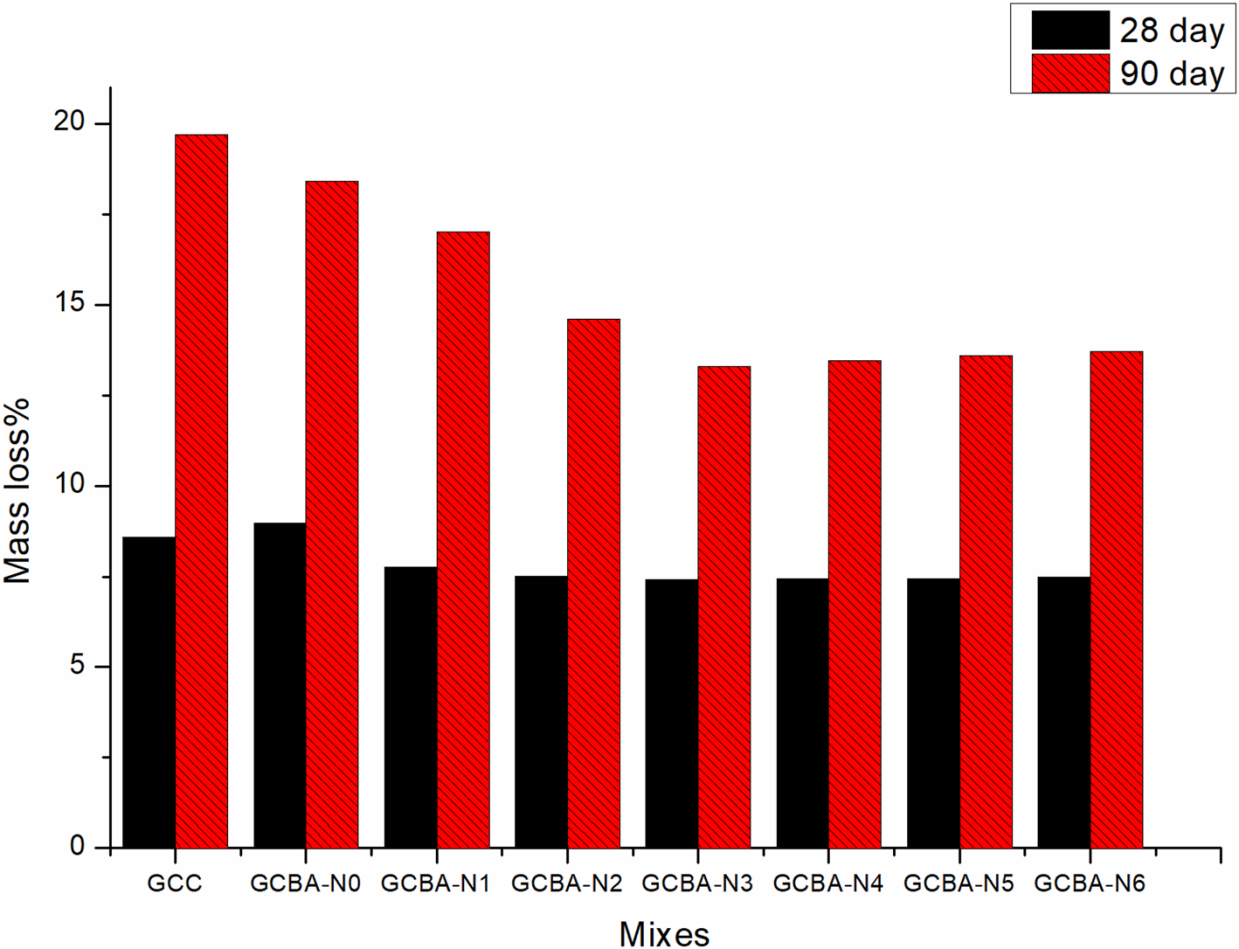
Mass loss % of geopolymer specimens.

Due to the attack of Na_2_SO_4_ on the geopolymer concrete specimen, the mass of the concrete specimen reduced gradually as the duration of immersing was increased. The effects of the Na_2_SO_4_ attack on the mass loss of geopolymer concrete are shown in Fig. 6. It was found that as the percentage of nano silica increased, the amount of mass loss decreased gradually. When bagasse ash was added to the binary mix of fly ash and GGBS the percentage of mass loss of ternary mix was noted as 2% less than the binary mix. But when the addition of nano-silica was introduced to the concrete mixture, the percentage of mass gradually decreased. When 4% of nano silica was added to the ternary concrete mix, the percentage of mass loss was 6%. The improvement in durability and mechanical properties of geopolymer concrete having nano-silica was achieved due to its high surface area and ultrafine grain size. The ultra fined nano silica filled the microscopic voids between the pozzolanic materials such as fly ash and GGBS and formed a dense structure. During the hydration process, the calcium aluminate hydroxide was produced and reacted with both nano silica and bagasse ash to form additional C-A-S-H gel. This secondary C-A-S-H gel improved the surface microstructure of geopolymer concrete and resisted penetrating sulfate ions into the concrete. It was found that further addition of nano-silica in concrete mixture did not change more in loss of mass and noted near about 6%. Because the geopolymer network structure’s Si–O–Al/Si bonds could be broken by an acid attack. Geopolymer concrete specimens cured at oven temperature might be less dense, which could make it easier for acids to move through their matrix, which would give them more surface area to do damage. The addition of bagasse ash up to 10% increased the amount of reactive phase in bagasse ash, which made it more advantageous. Because the inclusion of GGBS enhances the thermal curing temperature by producing internal heat during hydration and dissolution of geo-polymerization, a very important binder phase was formed during this step of the process. GCBA-N6 was more resistant to acidic media than GCC, which was why it had a better and denser matrix.

### In-elastic neutron scattering

**Figure 7:**
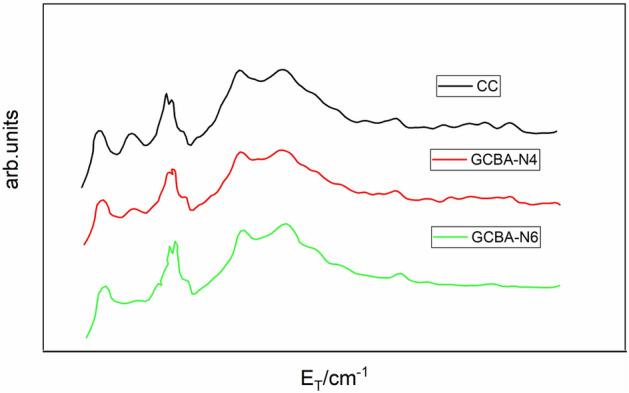
Inelastic neutron scattering spectra for different mixes of geopolymer concrete.

Inelastic neutron scattering (INS) is an effective approach to study the vibrational properties of substances and gives knowledge about the bonding properties of concrete. This method makes use of the different behavior between free atm and bound atom in the scattering method and uses the gap between the momentum and energy transfer to assemble the vibrational states of the system. A small piece of concrete was taken from each mix and ground into powdered form and produced a paste by adding water. It was spread by taking approximately 8 g of mixed ingredients in a thin 30 × 60 mm layer on an aluminum pan. After weighing it was immediately sealed by an indium gasket. Spectra were taken on an interval of 35 min and were obtained continuously for the first 48 h, then at regular intervals for samples cured at 20 °C. The device was tuned to a scattering angle of 90° with an energy resolution of 3 meV. Reference spectra were measured by placing 3.0 g of pure CH powder in the same cell and constraining it to the same transverse dimension as the sample material. The inelastic neutron spectra obtained from different geopolymer concrete mixes are shown in Fig. 7. The most obvious feature was found for mix GCBA-N6 mix as a sharp peak centered at 41 meV. The GCBA-N6 mix possessed high alkalinity materials due to the production of secondary CASH gel resulted in the oscillation of the OH groups with the high mode of intensities. The nano silica particles played a main role in the production of the secondary CASH gel. The absolute amount of formation of CH for GCBA-N6 mix during the entire hydration process by measuring the intensity of the peak as 41 meV which was a function of hydration time. The peak of traditional concrete was found as the lowest value because the rate of hydration process was slower than the nano silica based geopolymer concrete mixes. The peak value of GCBA-N4 and GCBA-N6 increased at a higher rate than CC. The inside temperature of traditional concrete might be decreased due to the slow rate of hydration. Hence, the onset of CH formation in traditional concrete might be systematically delayed with decreasing temperature. This method was based on the idea that a fixed (solid) material’s elastic spectrum was significantly different from a mobile (liquid) material’s. Such mobile scatterers caused an elastic spectrum broadening that had grown with increasing mobility. It was simple to distinguish between a spectrum’s fixed and mobile contributions, allowing one to estimate the proportion of energy that comes from solid matter and the remainder from mobile, liquid-like material. In hydrogenous materials, hydrogen tended to dominate neutron measurements because of its incredibly large incoherent scattering cross-section. The method used in the present study allowed us to concentrate on the water and identify the percentage of molecules that are liquid-like on a neutron time scale of ≤ 10^(−10)^ s and the percentage that has chemically reacted to form CH or become “bound” in the C-A-S-H gel-like structure. From the present research, it was found that, as the percentage of nano silica was increased the percentage of C-S-H gel-like structure increased rapidly and a “bound” like structure formed. Hence the peak of INS spectra for CC was found a lower value and a higher value was found in the GCBA-N6 mix. A new calcium silicate hydrate species that exhibited strong peaking corresponding to the Ca–OH bond, and good coordination between nano silica, sugarcane bagasse ash and fly ash had been developed. The calcium silicate hydrated phase’s molecular and IR spectra both exhibited a peak in that area, which was connected to the translatory movement of calcium hydroxide particles caused by the development of mesopores in sugar cane bagasse ash based concrete i.e. CC to GCBA-N6.Again, the bound hydrogen ratio is the ratio of the initial number of C_3_S molecules to the number of atoms that have become bound. Because the measured quantity contains both structural water and hydroxyl groups, it can be referred to as “bound hydrogen” rather than “bound water”. The higher percentage of nano silica absorbed more amount of water on its surface, which was utilized in the formation of further C-S-H gel and the formation of more amount of hydroxyl groups. Hence the bound hydrogen was found to double in mix GCBA-N6 mix than the control geopolymer concrete (CC). Since the water-carrying capacity of GCBA-N6 was more than other geopolymer concrete a general tendency was noticed for a higher fraction of hydrogen to become bound. Since more amount of water was absorbed by nano silica and sugar cane bagasse ash, the heat evolved during hydration process was found as lower value. It was noticed that the hydrogen distribution expressed as a function of time as the proportional number of moles of hydrogen atoms associated with each phase. It is evident that as temperature had risen, there was an increase in the amount of unreacted free water. Similar to this, the water content of the C-A-S-H phase, which was formed at higher temperatures, was systematically decreasing for mix of CC and GCBA-N4. The quaternary mix of supplementary cementitious materials which were used for binding materials of geopolymer concrete proved as the best combination for preparing geopolymer concrete and improved the microstructure of concrete significantly. As the percentage of nano silica was increased in geopolymer concrete the peak value inelastic neutron scattering was increased gradually by showing the high thick interfacial transition zone.

### X-ray diffraction test

The phenomenon of interactions between the incident X-ray beam and the electrons of the atoms of a material component, also known as coherent scattering, is represented by X-ray diffraction (XRD). The technique entails applying radiation to a sample and detecting the photons that are diffracted and make up the diffracted beam. An atom acts as a scattering center when an X-ray beam of a particular frequency focuses on it. The atom vibrates at the same frequency as the incident beam and spreads out in all directions. This incident beam will experience constructive interference in some directions when the atoms are arranged in a lattice, and destructive interference in others. When the path difference between a scattering beam’s successive planes is equal to an integer number of wavelengths, constructive interference of the scattered radiation results.

All the concrete samples were grinded to prepare powdered XRD samples. To reduce the sample loss during grinding and to reduce the structural damage to the phases in the sample, they were typically ground under a liquid medium like ethanol or methanol. The ground samples were crystalline powders that have been pressed into the sample holder, had a smooth surface, and were held in at a 45° angle. The film was arranged in a circle around the specimen, which was in the center of the camera. Diffraction occurred and distinctive X-rays were released in conical sections that intersected when a monochromatic X-ray beam was pointed at the specimen and utilized various arcs to expose the film. These arcs appeared as lines when the film was flattened. The distance on the X-ray film between the beam exit and entrance was kept at a 90° Bragg angle. The Bragg angle of each defining line in the film was calculated using the following subsequent ratio.

$$\frac{{S}_{i}}{{S}_{n}}= \frac{{\theta }_{i}}{{\theta }_{n}},$$

where $${S}_{i}=The\, distance\, from\, the \,exit\, to\, the \,line\, of\, intersect$$, $${S}_{n}=The\, distance\, between \,exit \,and \,entrance \,of\, the\, X-ray\, beam$$, $${\theta }_{i}=Incident\, angle\, (Bragg\text{'}s\, angle)$$, $${\theta }_{n}=Angle \,of \,the\, refracted \,ray$$.

When the refracted Bragg angle was located for each sample, the sample’s crystalline structure was ascertained by considering the crystal’s geometry.

**Figure 8:**
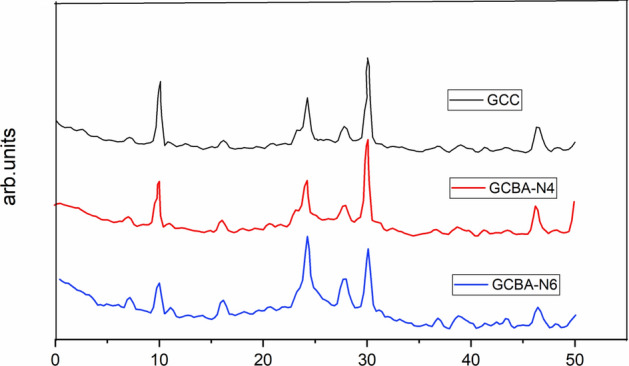
XRD test results for different mixes of geopolymer concrete.

The test data of XRD were collected from CuKα radiation of a diffractometer operated at 45 kV and 40 mA. Measurements were taken by making an incident of an X-ray beam on a flat-plate of Bragg–Brentano θ–2θ geometry. Air scatter was reduced with a beam knife and a 1° receive anti-scatter slit was placed in the path of the diffracted beam. A linear position sensing X-ray detector X’Celerator with a length of 2.212° as a 2θ was used for data collection. An angular range of 8° to 60° as 2θ with a 0.017° as 2θ step size was measured from the scanning of all mixes. Fine powder samples of different mixes of concrete were prepared using the backload method and repacked between repeated measurements to minimize the effect of favored orientation. The XRD diffraction test was conducted for evaluation of the crystalline phase and its amount at harden stage after 90 days hydration period. The XRD configuration of traditional concrete and ternary mix of modified concrete are shown in Fig. 8. The configuration hardened stage for both control and ternary based concrete at 90 days of curing was analyzed from X-ray diffraction taste and some important crystalline phases were identified. They were quartz, calcite, phlogopite, portlandite, andradite, biotite and their crystalline phases content was 18%, 10%, 6%, 1%, 1.5% and 5% respectively for GCBA-N6. The 2θ angle for quartz, calcite, phlogopite, portlandite, biotite is 34.73°, 30.30°, 24.27°, 14.21°, 17.32° and 22.24° respectively for CuKα. From the XRD analysis, it was identified as nano silica blended concrete from the mineral composition, which was showing a higher percentage of quartz. The portlandite crystals were decreased upto 1% for ternary concrete and up to 3% for traditional concrete. The increase in hydration product decreased the amount of portlandite in the GCBA N6 mix. As a result, the microstructure of ternary mixed concrete was improved when 6% of nano silica was introduced, which improved the durability and mechanical properties of concrete. The production of more hydration products filled the capillary pore spaces were the main causes of the strength enhancement. The peak of portlandite was decreased at the later period of the hydration.

The presence of C-A-S-H gel was detected in samples containing a mixture of fly ash slag and sugar cane bagasse ash as a precursor from the long peaks that represented the amorphous calcium silicate hydrate matrix. The Calcium Oxide peaks were visible in the XRD results for GCC, GCBA-N4, and GCBA-N6, which was equivalent to the case of SG 100 cured at 40 °C. This occurrence suggests that calcium aluminate silicate hydrate was forming as a result of the hydration of GGBS with nano-silica in the microstructure of the polymer product. The hydration of GGBS with nano silica was thought to resemble the hydration of Portland cement. With a different Ca/Si ratio than control geopolymer concrete (GCC), it led to the formation of a calcium silicate hydrate matrix. This hydration occurred concurrently with the polymerization processes connected to the alkali activation of fly ash and sugar cane bagasse ash. The formation of hydroxy sodalite, a low silica zeolite (Na6(Si_6_Al_6_O_24_)∙8H_2_O), was found with a distinguishing characteristic between the control geopolymer concrete sample and ternary mixed geopolymer concrete sample with nano silica. This was to be expected as zeolites like hydroxy sodalite were known to be produced during the hydrothermal NaOH treatment of sugar cane bagasse ash with nano silica. It should be noted that the geothermal silica sample (Fly Ash + GGBS + SCBA) exhibited a lot of major peaks. A few numbers of minor peaks were found in mix samples GCC, and GCBA-N4 in areas where sodalite formation was only minor. If the silica disintegrated gradually and the silicate species in solution did not move far from the surface of the particle, this might happen. As a result, some areas were silica-rich i.e. in the GCBA-N6 sample while others were silica-deficient i.e. in the GCC sample. The low silica crystalline hydroxy sodalite phase might form in the silica-deficient gel while the phase-separated system was bound by the higher silicate content X-ray amorphous phase.

## Conclusions

The effect on the strength and durability of geopolymer concrete composites with different amounts of nanoparticles was studied. Following are the findings of this study.

(i) The performance of geopolymer concrete prepared from a ternary combination of pozzolanic materials (i.e. fly ash, GGBS, and bagasse ash) was better than the binary combination (i.e. fly ash and GGBS) based geopolymer concrete. The addition of bagasse ash prepared secondary C-A-S-H gel, which improved the strength and durability properties.
(ii) The addition of nano-silica to the ternary combination-based geopolymer concrete up to 6%, improved the packing density and accelerated the performance at a faster rate.
(iii) As the content of nano silica was increased up to 6%, the consistency of geopolymer concrete was increased. The ball bearing action of nano silica improved the mobility of geopolymer concrete showing the highest slump value.
(iv) The strength and durability of the nanoparticles incorporated in geopolymer concrete specimens got the better result as the nanoparticle’s dosage varied from 1 to 4%. The geopolymer concrete composite that was made by a ternary combination of fly ash, GGBS, and sugar cane bagasse ash with 4% nanoparticles had shown the best test results for both compressive strength and split tensile strength after 7-days and 28-days curing.
(v) RCPT values of ternary combination geopolymer concrete with 4% nano-silica were very low for both 28-days and 90-days curing. The penetration of coulomb charge in the nano-silica mixed geopolymer concretes was observed lesser value than the binary combination of geopolymer concrete. The reduction of chloride ion penetration might be due to the incorporation of spherical particles of nano-silica, which improved the packing density of concrete.
(vi) Water absorption value of the ternary combination of geopolymer concrete reduced with increasing nano silica content. This happened due to the combined action of acceleration of the hydration process and pore fillings by extra C-A-S-H gel by which the geopolymer concrete became more compact and denser.
(vii) The improvement of surface microstructure in a ternary combination of geopolymer concrete with 4% or above of nano silica was too appreciable. All the mixes containing above 4% of nano silica protected geopolymer concrete from sulphate attack and reduced the percentage of mass loss in a satisfactory manner.

## Data Availability

The data used for this study are included in the manuscript itself.
